# Allergic diseases of the skin and drug allergies – 2035. Allergen control in management of chronic urticaria/angiodema (CUA) in Kenya, East Africa

**DOI:** 10.1186/1939-4551-6-S1-P120

**Published:** 2013-04-23

**Authors:** Tula Bowry, Priya Sikand

**Affiliations:** 1 Allergy Clinic, Nairobi Diagnostic Laboratory., Private Practice, Nairobi, Kenya

## 

A retrospective study on effect of allergen control is reported in 258 patients with CUA attending the allergy clinic over 5 years. The anaphylactic cases were excluded but no other selection was done.

Full clinical data, atopy and diet result of skin prick test (SPT) for tropical aeroallergens (TAA) and local diets were recorded including information on use of food addictives. The patients were followed up for evaluation of symptom scores and need for drugs for at least 5-6 months during which prophylactic usage for drug treatment was discouraged.

The patients with microscopic AA reactions, they were educated about the distribution and practical control measures with handouts in English and Kiswahili to be followed after 5-6 weeks to evaluate the progress.

The food allergy cases underwent open elimination dose related food rechallenged (OFC) for confirmation before advising rehabilitation.

The results showed 221 (85.8%) of patients were adults with M:F ratio 1:1:7. 120/258 (46.5%) has clinical atopy but 20 others only had family members with asthma. Multiple aeroallergen sensitivity is common which was not a problem in relation to control measures.110/258 (42.6%) reacted to house dust mite (HDM) 80/258 (31%) to mold, 35 (13.6%) to grass/weed pollen, 28 cases (10.9%) to cockroaches and 19 (7.4) to pets 28/258 (10.8%) had food allergies which may be under reported as many patients detect them.

The pattern of food allergies were 7 for cow milk, 6 for beef, 5 for eggs, 4 for red beans 2 for goat meat and 1 for chicken, 1 for fish, 1 for soya and 1 for banana. 22/258 (8.5%) CUA from over indulgence in food addictives which occurred more often in single patients 18-36 years old.

In 30/258 (11.6%) there were other clinical causes such as 6 with intestinal parasites, 4 with HIV, 4 with aspirin + NSAID sensitivity, 1 with thyrodectomy and 2 with hypothyroidism and 4 with poorly controlled diabetes 2 with C.T disease, 4 with serious stress.

